# Targeted enzyme gene re-positioning: A computational approach for discovering alternative bacterial enzymes for the synthesis of plant-specific secondary metabolites

**DOI:** 10.1016/j.mec.2019.e00102

**Published:** 2019-09-09

**Authors:** Yuya Nakamura, Shuichi Hirose, Yuko Taniguchi, Yuki Moriya, Takuji Yamada

**Affiliations:** a School of Life Science and Technology, Tokyo Institute of Technology, 2-12-1 Ookayama, Meguro, Tokyo, 152-8550, Japan; b NAGASE R&D Center, Nagase & Co., Ltd, Kobe High Tech Park 2-2-3 Murotani, Nishi- ku, Kobe, Hyogo, 651-2241, Japan; c Database Center for Life Science, Joint Support-Center for Data Science Research, Research Organization of Information and Systems, Kashiwa, 277-0871, Japan; d PRESTO, Japan Science and Technology Agency, 4-1-8 Honcho Kawaguchi, Saitama, 332-0012, Japan; e Metabologenomics Inc, 246-2 Kakuganji, Tsuruoka, Yamagata, 997-0052, Japan

**Keywords:** Enzyme repositioning, Plant secondary metabolism, Reaction similarity, Nootkatone, Genomics

## Abstract

Plant-biosynthesised secondary metabolites are unique sources of pharmaceuticals, food additives, and flavourings, among other industrial uses. However, industrial production of these metabolites is difficult because of their structural complexity, dangerousness and unfriendliness to natural environment, so the development of new methods to synthesise them is required. In this study, we developed a novel approach to identifying alternative bacterial enzyme to produce plant-biosynthesised secondary metabolites. Based on the similarity of enzymatic reactions, we searched for candidate bacterial genes encoding enzymes that could potentially replace the enzymes in plant-specific secondary metabolism reactions that are contained in the KEGG database (enzyme re-positioning). As a result, we discovered candidate bacterial alternative enzyme genes for 447 plant-specific secondary metabolic reaction. To validate our approach, we focused on the ability of an enzyme from *Streptomyces coelicolor* strain A3(2) strain to convert valencene to the grapefruit metabolite nootkatone, and confirmed its enzymatic activity by gas chromatography-mass spectrometry. This enzyme re-positioning approach may offer an entirely new way of screening enzymes that cannot be achieved by most of other conventional methods, and it is applicable to various other metabolites and may enable microbial production of compounds that are currently difficult to produce industrially.

## Highlights

• We developed ‘bacterial enzyme gene re-positioning’ that is a method to search for enzyme genes based on reaction similarity.
• We identified candidate bacterial alternative enzymes for 447 plant-specific secondary metabolism reactions.
• An enzyme for synthesising nootkatone from valencene was discovered.

## Graphical abstract

**Figure:**
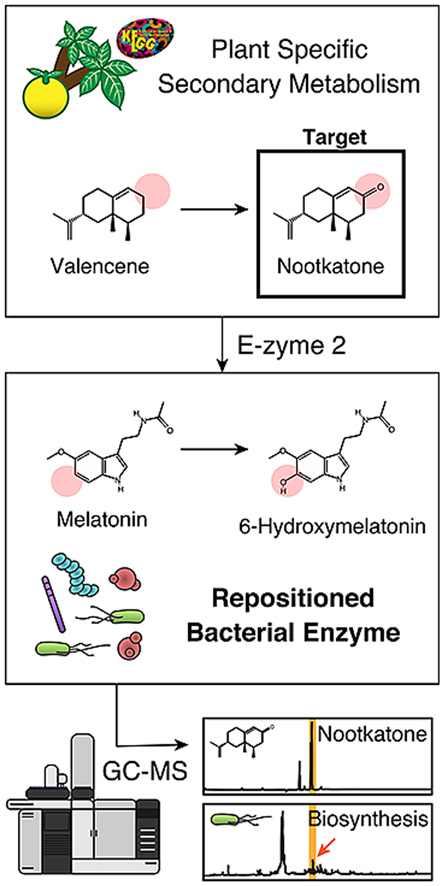

## 1 Introduction

Plants biosynthesis leads to the production of various compounds known as secondary metabolites, which are not required for plant growth, development, or reproduction, but are important for human daily life (Bourgaud et al., 2001). For example, rose, vanilla and star anise extracts are used for perfumes and medicines. However, these plant-specific secondary metabolites are often expensive because of the rarity of the compounds within the plants or the plants themselves. Some of these compounds are produced industrially, with direct extraction from the plants being the most common approach (Srivastava and Srivastava, 2007). However, there is the problem of resource exhaustion with this strategy, particularly for rare materials. Indeed, Ashtawarga plants as an Ayurvedic and allopathic medicine in India have been included among 560 plants appearing in the red list of endangered species (Virk et al., 2015). In addition, it can be difficult to obtain a stable supply due to climate change, and a simulative research has reported that the impact of climate change on the agricultural markets would amount to about one-sixth of total crop value (Costinot et al., 2016).

To overcome the challenges of extracting metabolites from plants, several means of synthesising metabolites artificially have been developed. Chemical synthesis is popular methods of stably obtaining metabolites that do not involve extraction from plants. Nonetheless, organic synthesis is not always readily possible, because plant secondary metabolites have complex structures and large molecular sizes. In such cases, organic synthesis requires many steps to obtain target metabolites from simple substrates, constituting a great hurdle for industrial large-scale synthesis of plant-specific secondary metabolites (Bruggink et al., 2003; Laine, 2017). On the other hand, microbial fermentation which is another method for synthesising metabolites enables the supply of stable plant-specific secondary metabolites without an environmental impact (Daniell et al., 2002; Velasco et al., 2000).

Nootkatone is one of the valuable plant-specific secondary metabolite and a factor for the characteristic flavor of grapefruit. In nootkatone production, organic synthesis is a general method, however it involves eight reaction steps (Laine, 2017) and requires the use of t-BuOOH, which is a dangerous substance (Julien and Wallace, 2008), or the massive use of heavy metals (MnO_2_), which has an environmental impact (Serra, 2016). Thus, a method was developed for producing nootkatone from valencene or hydroxyl valencene, which is inexpensive and easily available, using *Chlorella* (algae) (Asakawa et al., 2003). Furthermore, methods that employ laccase derived from microbes (Huang et al., 2001) and metabolism by *Mucor* (fungi) (Mihashi et al., 2008) and *Rhodococcus* (bacteria) (Ito et al., 2002) have been developed to produce nootkatone.

Microbial fermentation requires microbes capable of safely and efficiently producing target compounds, but such convenient microbes cannot easily be found. Therefore, techniques have been developed using genetic engineering to introduce genes encoding particular enzymes that can produce the target compound into safe microbes (Chang and Keasling, 2006; Lee and Kim, 2015; Peralta-Yahya et al., 2012). The candidates for these genes are generally obtained through searches of gene libraries using metagenomics (Jiang et al., 2011; Maimanakos et al., 2016; Popovic et al., 2017). For example, a screening of a library of P450 monooxygenase-encoding genes expressed in *Escherichia coli*, identified CYP109B1 from Bacillus subtilis that produces nootkatone from valencene (Girhard et al., 2009). However, identification of suitable genes using metagenomic screening usually requires experimentally screening libraries with a large number of genes.

Many previous studies have used computational searches based on gene sequence similarity to identify microbial genes homologous to plant genes encoding enzymes that are involved in the synthesis of secondary metabolites (Maimanakos et al., 2016; Montella et al., 2017). Nevertheless, such microbial genes were not found in most cases because synthesis of these secondary metabolites is almost exclusively a plant-specific process. Therefore, a new approach is needed to identify functional homologues of these plant genes in bacteria.

There are also methods for expressing transgenic plant genes in microbes (domain-level heterologous expression) and producing plant-specific secondary metabolites, yet this approach is extremely difficult because of decreasing expression level and enzyme activity due to differences in biological function (Flick et al., 2004; Kaur et al., 2018; Lambertz et al., 2014). In recent researches, novel techniques for heterologous expression are developing (Kaur et al., 2018; Lambertz et al., 2014), however, they are too insufficient to cover all of heterologous expression difficulties.

In this study, we developed a method to search for genes that encode enzymes based on similarity of enzyme reactions and not on gene sequences. In other words, we aimed to search for enzymes with similar metabolic reactions but with dissimilar gene sequences. Furthermore, in order to validate the approach, we discovered an enzyme for synthesising nootkatone from valencene that has no sequence similarity with the known plant enzyme.

## 2 Materials and methods

### 2.1 E-zyme2 search for bacterial alternative enzyme genes to substitute for plant-specific secondary metabolic enzymes

We used KEGG OC (Nakaya et al., 2013) database version 2015-03-19, which is a database of orthologue gene clusters linked to functional annotation (KO; KEGG Orthology), to screen for all plant-specific secondary metabolism reactions. From all reactions in the KEGG, we extracted 880 that only occur in plants and then searched for candidate bacterial genes whose products could replace these plant-specific enzymatic reactions, with specific reaction substrates and products, using E-zyme2, a web-based tool for searching for enzyme genes from a set of reaction substrates and products using enzymatic reaction similarity that is generally used to search for orphan enzymes with a known reaction but unknown gene (Moriya et al., 2016). To identify candidate bacterial enzymes for nootkatone biosynthesis, we used KEGG OC database version 2016-01-16.

### 2.2 Bacterial strains and culturing conditions

*Streptomyces lividans* TK64 (NBRC 15678), which was purchased from National Institute of Technology and Evaluation (NITE, Chiba, Japan), was used as the host organism for bioconversion. *S. lividans* TK64 was cultured in TSB medium (17 g/L pancreatic digest of casein, 3 g/L papaic digest of meal, 2.5 g/L glucose, 5 g/L sodium chloride, and 2.5 g/L dipotassium phosphate, BD® Tryptic Soy Broth (Catalogue no. 211825); BD, Heidelberg, Germany) supplemented with 50 μg/mL of thiostrepton at 28 °C and 200 rpm for 72 h.

*Escherichia coli* JM109 (Takara Bio, Shiga, Japan) was used as the host organism for constructing plasmid clones. *Escherichia coli* JM109 was cultured in LB medium (Nacalai tesque, Kyoto, Japan) at 37 °C for 16 h.

### 2.3 Plasmid construction

For constructing expression vectors, four genes derived from *Streptomyces coelicolor* A3(2) (GenBank No. NC_003888), *SCO5223*, *SCO3099*, *SCO3770* and *SCO3636* were cloned. The genomic DNA of *S. coelicolor* A3(2) was acquired by phenol-chloroform extraction. The coding sequences of four genes were amplified with primers (Supplementary Table 1) using the *S. coelicolor* A3(2) genomic DNA as a template. PCR was performed under the following conditions: 94 °C for 2 min, followed by 30 cycles of 98 °C for 10 s and 68 °C for 1.5 min (KOD-Plus-Neo; Toyobo, Osaka, Japan). The PCR products were purified and digested with SphI, except for the *SCO3636* PCR product, which was digested with EcoT22I. pIJ702D, a derivative of pIJ702SSMP from which *melC1* and *melC2* have been removed (Supplemental Fig. 1A), was also digested with StuI and SphI (or EcoT22I for *SCO3636*) (Comalada et al., 2005). The PCR products were ligated into the digested vector to produce the expression plasmids. The ligation reaction was incubated with Ligation Mix (Takara Bio, Shiga, Japan) at 16 °C for 1 h.

To construct the *SCO3770* partially-knockout vector, nucleotides 97 to 1090 of *SCO3770*, which encode an inactive form of the enzyme, were amplified with primers (Supplementary Table 2) using the *S. coelicolor* A3(2) genome as a template. PCR was performed under the following conditions: 98 °C for 30 s, followed by 30 cycles of 98 °C for 30 s, 55 °C for 30 s, and 68 °C for 1 min (PrimeSTAR® GXL DNA Polymerase; Takara Bio, Shiga, Japan). The PCR product was purified and digested with EcoRI and KpnI. pk18melC, a derivative of pk18mob carrying *melC1* and *melC2* was digested with the same restriction enzyme, and the fragments were ligated together (Supplemental Fig. 1B). The ligation condition was same as that in expression vectors.

### 2.4 Transformation

The p450 gene expression vectors were introduced into *S. lividans* TK64 via protoplast transformation according to Keiser’s method (Dang et al., 2018). Transformants were selected based on thiostrepton resistance.

Similarly, *SCO3770* was knocked out in *S. lividans* TK64 by introducing the pIJ702D:*ΔSCO3770* vector. Transformants were selected based on kanamycin resistance. To construct the complemented strain, protoplasts from *S. lividans* TK64 *ΔSCO3770* were transformed with pIJ702D:*SCO3770* as above.

### 2.5 Conversion of valencene into nootkatone by *Streptomyces*

Single colonies of transformants were pre-cultured in 5 mL TSB medium supplemented with 50 μg/mL of thiostrepton at 28 °C and 200 rpm for 72 h. A 1.5-mL aliquot of this pre-culture was inoculated into a baffled flask containing 50 mL SSMP medium (5% glucose, 0.8% K2HPO4, 0.05% MgSO4・7H2O, 0.5% yeast extract, 0.5% polypeptone) supplemented with 10 μg/mL of thiostrepton and incubated at 28 °C and 160 rpm. After 72 h, glucose and valencene (Catalogue no. 75056-10G-F; SIGMA-ALDRICH, Missouri, USA) were added at final concentrations of 10% and 0.025%, respectively. Cultivation was then continued for another 168 h.

### 2.6 Confirmation of nootkatone production

Following culture of the P450-overexpressing strains, cells were harvested from 2 mL whole broth by centrifugation at 14,000×*g* for 20 min. The cell pellet was resuspended in 1.5 mL ethanol, and 0.25 mL distilled water and 0.5 mL *n*-hexane were added to 1.0 mL of the supernatant and mixed by shaking. The *n*-hexane layer was collected by centrifugation at 14,000×*g* for 1 min. Nootkatone in the extract was analysed by GC/MS (GC-2010 and GCMS-QP2010 Plus, Shimadzu; Kyoto, Japan). The peak of nootkatone was at approximately 12.0 min. Measurements were performed under the conditions indicated in Supplemental Table 2. Purified purchased nootkatone (Catalogue no. N0920; Tokyo Chemical Industry, Tokyo, Japan) was used for nootkatone standard.

### 2.7 Quantification of nootkatone

Four strains (*S. lividans* TK64, *S. lividans* TK64/pIJ702D:*SCO3770*, *S. lividans* TK64 Δ*SCO3770*, and *S. lividans* TK64 Δ*SCO3770*/pIJ702D:*SCO3770*) were cultured, and cells were harvested from 2 mL whole broth by centrifugation at 14,000×*g* for 20 min. Next, 0.5 mL *n*-hexane was added to 1.0 mL of the supernatant and mixed by shaking, after which the *n*-hexane layer was separated by centrifugation at 14,000×*g* for 1 min. The cell pellet was resuspended in 1.5 mL ethanol, and 0.25 mL distilled water and 0.5 mL *n*-hexane were added to 1.0 mL of the supernatant and mixed by shaking. Nootkatone in both extracts was analysed by GC/MS (GCMS-QP2010 Plus; Shimadzu, Kyoto, Japan) under the conditions indicated in Supplemental Table 3. The amount of nootkatone was estimated based on the standard curve for β-caryophyllene (Catalogue no. C0796; Tokyo Chemical Industry, Tokyo, Japan).

### 2.8 Data analysis

We used TogoWS (Katayama et al., 2010) and KEGG API (Kanehisa et al., 2019) to extract data from KEGG and format the data. Python was used for summarising and visualising the enzyme search results. iTol4 (Letunic and Bork, 2016) was used to visualise the phylogenetic distribution of bacterial enzymes that could potentially replace plant-specific enzymes.

The amino acid sequence of the nootkatone biosynthetic enzyme was obtained from the UniProt database (Bateman et al., 2017). Homology searches of SCO3770 and nootkatone biosynthetic enzymes were performed using BLAST (Altschul et al., 1990) with default options. COBALT (Papadopoulos and Agarwala, 2007) was employed for the multiple alignment.

## 3 Results

### 3.1 Identification of candidate bacterial enzyme genes for plant-specific secondary metabolite biosynthesis

**Fig. 1:**
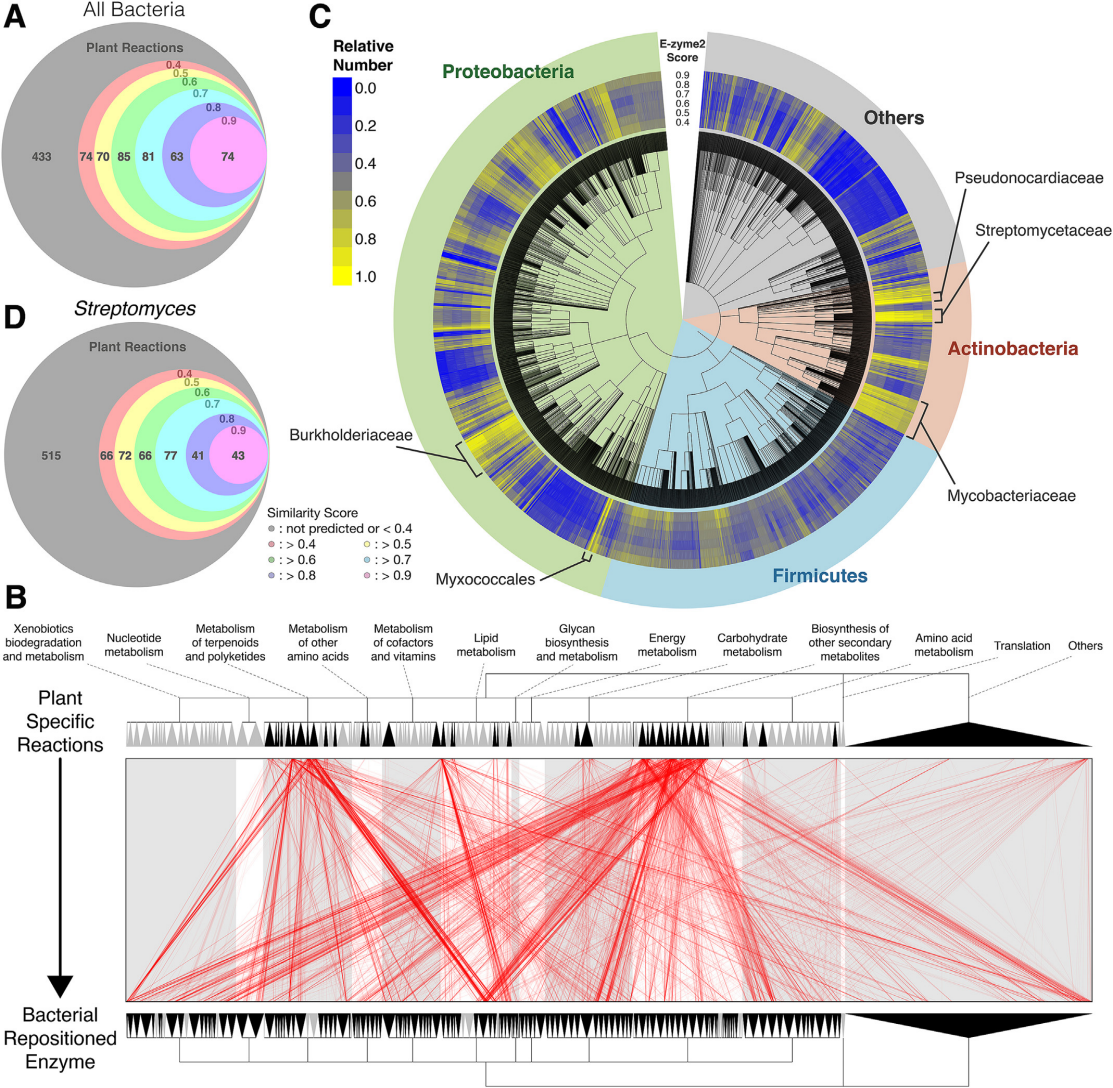
The validation of the screening range by a reaction similarity-based method. A, D: Venn diagrams showing the number of reactions for which E-zyme2 detected enzymes in either any bacterium (A) or Streptomyces coelicolor A3(2) (D) as alternative enzyme candidates from 880 plant-specific secondary metabolism reactions. The numbers at the tops of the circles represent reaction similarity scores based on E-zyme2, and the numbers at the centre of each region represent the number of reactions detected over that reaction similarity score. B: We classified KEGG REACTION functionally based on KEGG BRITE and visualised the functional distribution of plant-specific secondary metabolism reactions and bacterial alternative enzyme candidates. Plant-specific secondary metabolic reactions and bacterial alternative enzyme candidates are connected by a red line. The top and bottom trees represent functional classification trees; black colour indicates a reaction pathway in which the reaction or enzyme candidate exists, and grey colour indicates a reaction pathway in which the reaction or enzyme candidate does not exist. C: Distribution of the number of alternative enzyme candidates found in each bacterial strain. The central dendrogram represents the bacterial phylogenetic tree, and the surrounding heatmap shows the relative number of reactions that each bacterial strain is predicted to substitute. (For interpretation of the references to color in this figure legend, the reader is referred to the Web version of this article.)

To determine whether bacterial enzymes could be identified that perform similar functions to plant enzymes involved in secondary metabolite production, we first obtained 880 substrate/product compound pairs of plant-specific metabolic reactions from KEGG database (Kanehisa et al., 2019), which is an integrated database of genes, metabolic pathways and metabolites. Next, we used the 880 substrate/product pairs as a query for E-zyme2 (Moriya et al., 2016) to search for bacterial enzyme genes that appeared to replace the plant enzyme. E-zyme2 searches for “similar reactions” in KEGG using a substrate/product compound pair as the query and returns orthologue gene candidates and reaction similarity scores. The “similar reactions” does not mean that a substrate/product pair of query is chemically and structurally similar to substrate/product pairs of predicted enzyme but that the patterns of chemical changes occurring during the reactions (referred to as “RDM” patterns) is similar (Moriya et al., 2016). This tool enabled us to search for bacterial alternative enzyme genes for plant-specific metabolic reactions based on reaction similarity and not gene sequence similarity. E-zyme2 identified 447 of 880 plant-specific metabolic reactions as a reaction that might be catalysed by bacterial enzymes, with a similarity score ≥0.4 (Fig. 1A, Supplementary Table 4). Of these 447 reactions, 41 reactions involved orphan enzymes in plant (Supplementary Table 5). In addition, 19 reactions of these belong to flavone-related metabolic pathways.

Metabolic reactions in KEGG have a functional hierarchical structure consisting of reactions, pathway and biological functional category (Kanehisa et al., 2019; Uchiyama et al., 2015). To confirm the multifunctional ability of bacterial enzymes, we projected target plant reactions and the native reactions of the detected bacterial enzymes by extrapolating the plant reactions onto the KEGG functional hierarchy. As a result, bacterial alternative enzyme candidates for plant reactions were different from the query plant reactions (Fig. 1B).

To identify bacterial strains that contain a high proportion of candidate enzyme genes for producing plant secondary metabolites, we aggregated the number of reactions for each bacterial species and visualised the phylogenetic distribution using iTol4 (Letunic and Bork, 2016) (Supplementary Table 6, Fig. 1C). As shown, there was bias in the number of alternative gene candidates among taxa, with bacteria belonging to Actinobacteria and Proteobacteria possessing the highest number of alternative gene candidates that might replace genes involved in plant-specific metabolism. In particular, Streptomycetaceae, Pseudonocardiaceae, Mycobacteriaceae, Myxococcales and Burkholderiaceae harbours more alternative gene candidates compared to the other bacteria. Of these bacteria, the *Streptomyces coelicolor* A3(2), which is a representative *Streptomyces* species and generally used for enzyme screening, exhibited 365 reactions with a similarity score ≥ 0.4, and 43 reactions with a similarity score ≥ 0.9 (Fig. 1D). Therefore, we focused on sco for subsequent experiments and analysis.

### 3.2 A *Streptomyces coelicolor* A3(2) gene product metabolises valencene to nootkatone

To validate the ability of our enzyme re-positioning approach to accurately predict bacterial gene products that can substitute for plant-specific enzymes, we assayed enzymatic activity for *S. coelicolor* gene products predicted to catalyse synthesis of nootkatone from valencene. The reason for choosing this reaction was that the product, nootkatone, was highly valuable and the substrate, valencene, was easily available. In addition, the reaction similarity scores for the candidate genes in S. coelicolor was relatively high compared to all of the reactions that were assessed, as shown in Supplementary Table 6.

We detected four *S. coelicolor* genes (*SCO5223*, *SCO3099*, *SCO3636* and *SCO3770*), which belong to the CYP170A, CYP107U, CYP107P and CYP107T subclasses of the P450 family, respectively, as alternative enzyme candidates for nootkatone biosynthesis according to their similarity scores (Table 1). Our enzyme re-positioning approach is based on reaction similarity, which enabled us to search for genes encoding alternative bacterial enzymes without sequence similarity to the relevant plant enzymes. Therefore, to confirm that these four genes was discovered based on the sequence independence of our approach, we performed a multiple alignment of these four genes by BLAST and the three nootkatone synthetases reported previously (Cankar et al., 2011; Fraatz et al., 2009; Girhard et al., 2009) (Table 2). For the eukaryotic-derived genes *E1B2Z9* (*Cichorium intybus*) and *B8ZIU7* (*Pleurotus sapidus*), the total scores (BLAST bit score) compared to four genes were low, less than 54.6 and identity was also low, less than 30%. For the prokaryotic-derived gene U5U1Z3 (*Bacillus subtilis*), the BLAST bit score was 99–229, and some homology with four genes was observed, even though identity was only 24–36%. Thus, it could never be considered that these scores were top hits for sequence similarity based screening.

**Table 1:** Nootkatone biosynthesis enzyme candidates.

Gene name	Score	KEGG OC (ver. 2016-01-16)	Definition (RefSeq)	KEGG Orthology
*SCO5223*	0.646	OC.66774	cytochrome P450	K12645: epi-isozizaene 5-monooxygenase
*SCO3099*	0.411	OC.19251	cytochrome P450 hydroxylase	–
*SCO3636*	0.411	OC.19251	cytochrome P-450 hydroxylase	K00493: unspecific monooxygenase
*SCO3770*	0.411	OC.19251	cytochrome P450 oxidoreductase	–

**Table 2:** Protein sequence alignment of nootkatone biosynthesis enzyme candidates with known nootkatone biosynthesis enzyme by BLAST.

Query	Target	Max Score	Query Cover	E value	Identity
SCO3770	U5U1Z3	202	82%	2.00E-65	36%
	E1B2Z9	44.7	42%	7.00E-09	26%
	B8ZIU7	22.7	28%	0.057	26%
SCO3636	U5U1Z3	204	93%	9.00E-66	30%
	E1B2Z9	20.8	25%	2.20E-01	30%
	B8ZIU7	N.A.	N.A.	N.A.	N.A.
SCO3099	U5U1Z3	229	91%	3.00E-75	33%
	E1B2Z9	54.7	75%	6.00E-12	23%
	B8ZIU7	N.A.	N.A.	N.A.	N.A.
SCO5223	U5U1Z3	99	87%	3.00E-26	24%
	E1B2Z9	42.7	61%	4.00E-08	21%
	B8ZIU7	N.A.	N.A.	N.A.	N.A.

N.A.: Not Aligned.

**Fig. 2:**
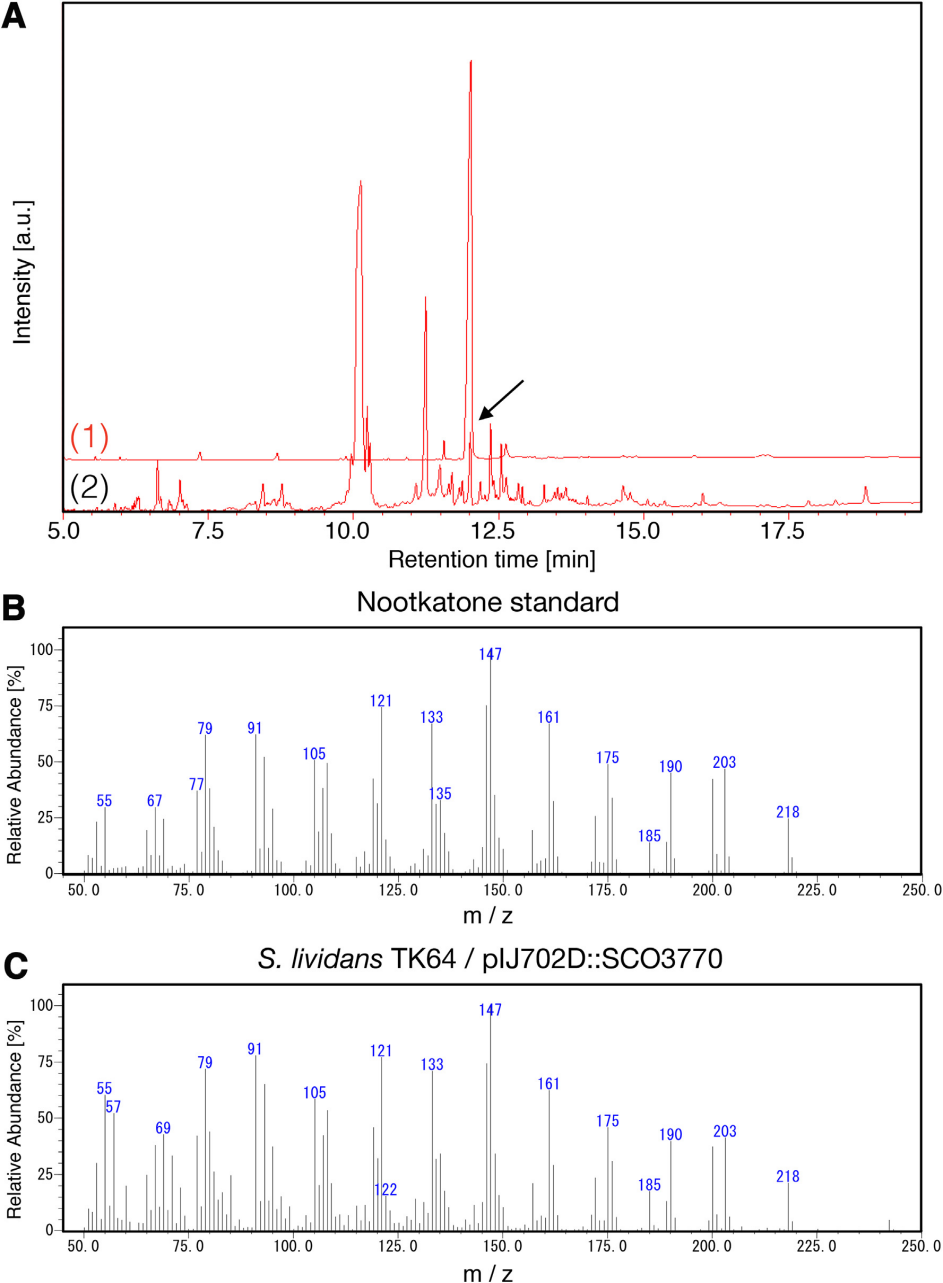
Gas chromatography-mass spectrometry analysis of the *SCO3770*-overexpressing strain. A: Gas chromatographic chart of the purified purchased nootkatone standard (1) and *S. lividans* TK64/pIJ702D:*SCO3770* (2). B: Mass spectrometry result for the nootkatone standard. C: Mass spectrometry result for *S. lividans* TK64/pIJ702D:*SCO3770*.

To assess the activity of these enzymes, we overexpressed them in *S. lividans* TK64 using p450 overexpression vectors. *S. lividans* TK64 was a model species and a closely-related to S. coelicolor, and commonly used in genetic engineering (Comalada et al., 2005; Miyashita et al., 1991; Saha et al., 2017). After these overexpressing strains were cultured for 72 h, 0.025% (w/v) valencene was added and bio-conversion of valencene to nootkatone was conducted for 168 h. After that, the culture supernatant and cell pellet were extracted with hexane solvent because both valencene and nootkatone are lipophilic compounds and the bio-conversion by P450 was thought to occur near the cell membrane. The nootkatone in hexane was detected by gas chromatography-mass spectrometry (GC-MS). A peak of nootkatone equal to a peak identified by the standard nootkatone was observed in the *SCO3770*-overexpressing strain (Fig. 2A), and this fraction was analysed by MS. The pattern of peaks was consistent with that of a nootkatone chemical standard (Fig. 2B and C). Thus, we concluded that the *SCO3770*-overexpressing *S. lividans* TK64 strain effectively metabolised nootkatone from valencene.

### 3.3 Characterisation of *SCO3770*

**Fig. 3:**
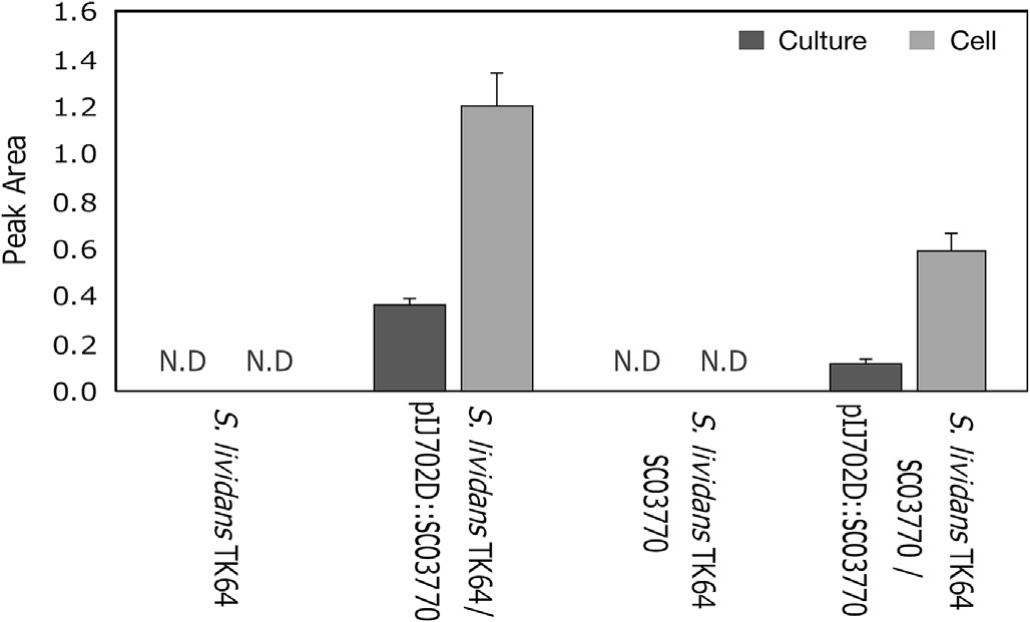
Characterisation of SCO3770. A: The peak area on the vertical axis shows the production of nootkatone in wild-type *S. lividans* TK64 and overexpressing strain, and after *SCO3770* deletion and complementation. N.D. represents “Not Detected”. B: Multiple alignment of amino acid sequences of SCO3770 and previously known nootkatone biosynthetic enzymes. Identical amino acids are shown as the same colour. “Max Score” and “Identity” represent the maximum score and identity of the sequence homology search result using BLAST.

To confirm the involvement of *SCO3770* in nootkatone biosynthesis, we performed a genetic complementation assay because *S. lividans* TK64 had a homologous gene of *SCO3770* which has two amino acids mismatch with SCO3770 (D121G, G371D). First, *SCO3770* overexpressing (*S. lividans* TK64/pIJ702D:*SCO3770*), genomic *SCO3770* homolog partially-knockout (*S. lividans* TK64 Δ*SCO3770*) and *SCO3770* complementation (*S. lividans* TK64 Δ*SCO3770*/pIJ702D:*SCO3770*) strains were constructed. Next, these three strains and wild-type *S. lividans* were cultured in medium containing valencene for 168 h, the culture supernatant was extracted with hexane solvent, and the nootkatone in hexane was detected by GC-MS (Fig. 3). Although the wild-type strain did not produce nootkatone, *S. lividans* TK64/pIJ702D:*SCO3770* did. *S. lividans* TK64 Δ*SCO3770* did not produce nootkatone, but nootkatone production was restored in the *SCO3770* complementation strain (*S. lividans* TK64 Δ*SCO3770*/pIJ702D:*SCO3770*). These results indicate that *SCO3770* is involved in the conversion of valencene to nootkatone.

## 4 Discussion

In this study, we developed ‘bacterial enzyme gene re-positioning’ that is a method to search for enzyme coding genes based on similarity of enzyme reactions, and identified candidate bacterial alternative enzymes for 447 plant-specific secondary metabolism reactions. In addition, an enzyme for synthesising nootkatone from valencene, SCO3770 was discovered by our approach, and SCO3770 had low sequence homology with existing enzymes. This result showed that our ‘bacterial enzyme gene re-positioning’ approach has a novel enzyme screening space which is not considered by sequence based similarity approach, and it may enable us to produce compounds that are currently difficult to produce industrially by microbial fermentation.

Our bacterial enzyme gene re-positioning approach detected candidate bacterial alternative enzymes for 447 plant-specific secondary metabolism reactions, and 41 of 447 (9.2%) of the plant-specific secondary metabolism reactions for which re-positioning candidates were identified involved “orphan enzymes”, whose reactions are reported but the genes are not discovered (Yamada et al., 2012). Although we call this method “enzyme re-positioning”, we can identify genes that encode alternative enzymes for target reactions, even if the target enzymes are orphan enzymes. It is pretty obvious, but sequence based enzyme genes screening required the gene sequence. However, E-zyme2 was originally designed to be used as a tool for orphan enzyme discovery (Moriya et al., 2016). Thus, it can also be used to search for candidate enzymes using only a substrate and product pair.

Of the plant-specific orphan enzymatic reactions for which we found alternative enzymes in this study, about half of the 19 reactions belonged to flavone-related metabolic pathways. Flavone-related compounds reportedly have anti-tumour (Cincin et al., 2014; Dória et al., 2016; Martino et al., 2016; Sung et al., 2016; Yan et al., 2017) and anti-inflammatory (Comalada et al., 2005; Dang et al., 2018) effects and high medical usefulness. In particular, apigenin has been reported to function in cancer prevention by inducing apoptosis and autophagy (Sung et al., 2016), and it is expected to act as an anticancer agent (Yan et al., 2017). If alternative enzymes enable inexpensive production of these types of compounds, this would be of great benefit in the research and development of new anti-tumour drugs.

Our result of phylogenetic distribution analysis for discovered candidate bacterial alternative enzymes indicated that the bacterial enzyme candidate genes clustered into a few taxa. The bacterial enzyme candidates were phylogenetically biased, with Pseudonocardiaceae, Streptomycetaceae, Mycobacteriaceae, Myxococcales and Burkholderiaceae possessing more alternative enzyme candidates than other taxa. Since Streptomycetaceae and Burkholderiaceae have particularly many secondary metabolic functions among all of bacteria, it is possible that such bacterial secondary metabolism related enzymes have been detected as alternative candidates for plant secondary metabolism. Moreover, all these bacteria live in soil (Kim et al., 2015; Walters et al., 2018), and since Pseudonocardiaceae, Streptomycetaceae, Mycobacteriaceae, and Burkholderiaceae is frequently detected in the soil near the roots of plants (Child et al., 2007; Salles et al., 2004; Santos-Medellín et al., 2017; Wang et al., 2016; Yeoh et al., 2017), these bacteria may metabolize plant secondary metabolic compounds using the candidate genes that we found. In any case, the soil metagenome is likely to be an important resource source for the synthesis of plant secondary metabolic compounds.

In this study, we applied this method to plant secondary metabolism, however the scope of application of this method is not limited to plant reactions. It is possible to search for the enzyme reactions of all organisms. Not only that, it is possible to search for reactions in which no enzyme has been found if we have only a substrate-product pair. Therefore, applying this method may enable us to flexibly search for the enzyme genes for the desired reaction. This method may be used to search for enzymes for producing industrially valuable compounds from inexpensive compounds without considering whether the reactions are catalysed by the enzymes. the reactions.

## 5 Conclusions

Taken together, we developed a novel method, bacterial enzyme gene re-positioning, to efficiently screen bacterial alternative enzymes for plant-specific secondary metabolism based on reaction similarity and found a nootkatone biosynthetic enzyme and many alternative enzyme candidates for other reactions. This bacterial enzyme gene re-positioning method may offer an entirely new way of screening enzymes that cannot be achieved by most of other conventional methods. Overall, this method will enable the production of compounds that are difficult to synthesise. In the future, the discovery of new alternative enzymes through application of this methodology will enable microbial fermentation-mediated mass production of various compounds.

## Financial disclosure

This research did not receive any specific grant from funding agencies in the public, commercial, or not-for-profit sectors.

## Author contributions

Y. M., S. H. and T. Y. designed this study. Y. N. and Y. M. performed the bioinformatic screening and other bioinformatic analysis. Y. T. performed the bacterial culture and other experiments. T. Y. and S. H. provided senior guidance. Y. N. wrote the manuscript with assistance from other authors. All authors reviewed and approved the final version of the paper.

## Conflicts of interest

No conflict of interests is declared.

## Appendix A Supplementary data

Multimedia component 2 Multimedia component 2

Supplementary Fig. 1 Vector map. A: Schematic diagram of the pIJ702D used to express the P450-encoding genes. P450 genes were inserted between StuI site and EcoT22I/SphI site. *SSMP*pro, promoter region of metalloendopeptidase from *Streptomyces septatus*; *PLD*ter, terminator region of phospholipase D from *Streptomyces cinnamoneus*; rep pIJ101, replicon of *Streptomyces* from pIJ101; Tsr, thiostrepton resistance gene. B. Schematic diagram of the *SCO3770* partially-knockout vector. Δ*SCO3770*, region of position 97 to 1090 in *SCO3770*; melC1C2, *melC1* and *melC2* operon from *Streptomyces antibioticus*; Km, kanamycin resistance gene; oriT and oriV, replicon of *E. coli* 3781-478. Supplementary Fig. 1
