# Supplementary figures and images for: Targeted enzyme gene re-positioning: A computational approach for discovering alternative bacterial enzymes for the synthesis of plant-specific secondary metabolites
Source: Metab Eng Commun. 2019 Sep 9;9:e00102. doi: 10.1016/j.mec.2019.e00102 (PMC6838473; doi:10.1016/j.mec.2019.e00102)

**A**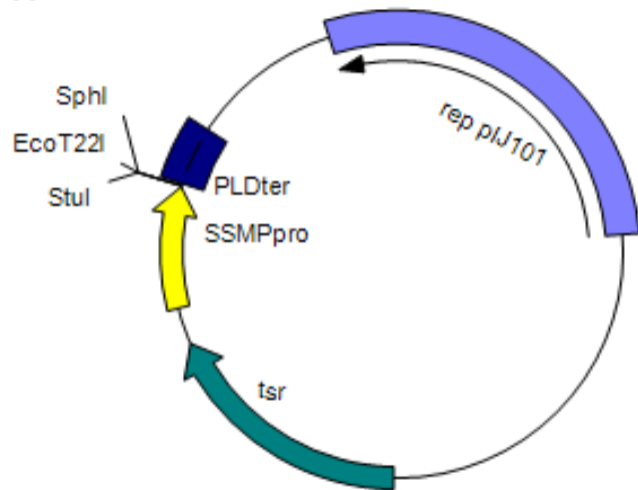**B**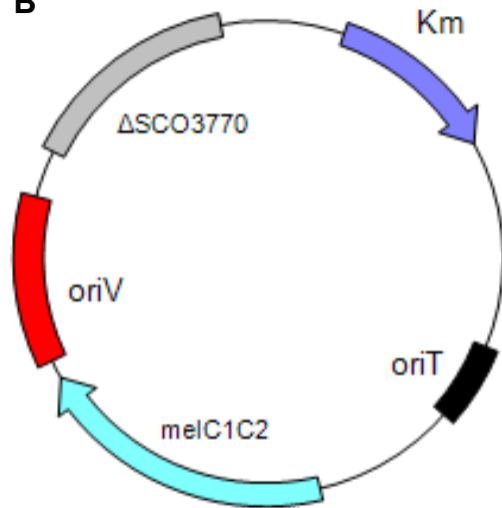

Supplement: Supplementary Fig. 1 — Vector map. A: Schematic diagram of the pIJ702D used to express the P450-encoding genes. P450 genes were inserted between StuI site and EcoT22I/SphI site. SSMPpro, promoter region of metalloendopeptidase from Streptomyces septatus; PLDter, terminator region of phospholipase D from Streptomyces cinnamoneus; rep pIJ101, replicon of Streptomyces from pIJ101; Tsr, thiostrepton resistance gene. B. Schematic diagram of the SCO3770 partially-knockout vector. ΔSCO3770, region of position 97 to 1090 in SCO3770; melC1C2, melC1 and melC2 operon from Streptomyces antibioticus; Km, kanamycin resistance gene; oriT and oriV, replicon of E. coli 3781-478. [file mmc3.pdf]
